# Experiences of being screened for intimate partner violence during pregnancy: a qualitative study of women in Japan

**DOI:** 10.1186/s12905-018-0566-4

**Published:** 2018-05-29

**Authors:** Yaeko Kataoka, Mikiko Imazeki

**Affiliations:** 1 St. Luke’s International University, Tokyo, Japan; 2 St. Luke’s International Hospital, Tokyo, Japan

**Keywords:** Intimate partner violence, Pregnancy, Screening experience, Qualitative study

## Abstract

**Background:**

Intimate partner violence (IPV) is physical, sexual or psychological violence by a current or former intimate partner. IPV threatens women’s health, and during pregnancy women are more vulnerable to violence. Therefore, IPV screening has been recommended during antenatal care; however, health care providers have expressed concern about the negative impact on women and therefore have been reluctant in conducting IPV screening. Consequently our objective was to investigate pregnant women’s experiences of reading and completing an IPV screening questionnaire.

**Methods:**

Semi-structured interviews were conducted with postpartum women who had received IPV screening during pregnancy to investigate their experiences using the IVP Violence Against Women Screen (VAWS). Qualitative data were analyzed based on content analysis.

**Results:**

A total of 43 women participated in this study. There were eight (18.6%) women positive for IPV screening during pregnancy. Content analysis for all participants revealed three themes: necessity, acceptability and optimality. ‘Necessity’ referred to benefits for women from IPV screening, and was supported by three categories: ‘redefining the relationship’, ‘promoting IPV awareness’ and ‘opportunity to initiate support’. ‘Acceptability’ of IPV screening was also supported by three categories: ‘comfortable’, ‘quickly completed’ and ‘difficulty’. ‘Optimality’ meant IPV screening during pregnancy was appropriate timing for women who had been screened as either positive or negative.

**Conclusions:**

The majority of women, including women experiencing IPV, had positive responses to IPV screening during pregnancy. Future diffusion of IPV screening requires safe environments for IPV screening and improved awareness of health care providers towards IPV.

**Electronic supplementary material:**

The online version of this article (10.1186/s12905-018-0566-4) contains supplementary material, which is available to authorized users.

## Background

Intimate partner violence (IPV) is defined as behavior by an intimate partner that causes physical, sexual or psychological harm, including acts of physical aggression, sexual coercion, psychological abuse and controlling behaviors [1]. The World Health Organization stated that violence against women is a major global public health problem and human rights concern. The study by the WHO [2] in ten different countries reported that 13%-61% of women had been abused by their intimate male partners. IPV is also a serious social problem in Japan. The Gender Equality Bureau Cabinet Office [3] in Japan conducted a national survey and found 15% of women experienced physical violence, 12% of women were assaulted psychologically by male partners and 7% of women experienced coercive sexual intercourse. In addition, the rate of IPV victims has not changed for over ten years since the government survey started in 1999. It is urgent that solutions be developed to eliminate violence against women in all countries.

Moreover, IPV towards pregnant women is a serious concern that the world faces. A systematic review indicated that prevalence of IPV during pregnancy ranged from 0.9% to 20.1% [4]. Research on Japanese pregnant women that applied the Index of Spouse Abuse (ISA) [5] found that 5% of women experienced IPV during pregnancy, and another survey showed that 1% of pregnant women had experienced physical intimate partner violence [2]. IPV threatens several aspects of health among pregnant women. These include not only physical injuries [6] but also psychological impairment such as postpartum depression [7], posttraumatic stress disorder [8, 9], bonding disorders [10] and suicidality [11]. Violence during pregnancy can result in pregnancy complications such as hypertension, vaginal and cervical bleeding, placental problems, severe nausea, and kidney infection [12]. In addition IPV during pregnancy affects the fetus and neonates, such as low birth weight, preterm delivery and neonatal death [13–15]. It may cause life-threatening results or the death of the mother and child [13, 15, 16].

In Japan, the Act on the Prevention of Spousal Violence and the Protection of Victims was promulgated in April 2001 in recognition of the high IPV prevalence and its adverse social and health outcomes in Japan [17]. This was the first law to indicate explicitly that spousal violence was a criminal act and the perpetrator must be punished by the criminal code. It also prescribed that prefectures establish and authorize at least one or more spousal violence counseling and support centers to take a central role in the support system for IPV victims in Japan. As a result of the Act, the number of institutions such as shelters, women’s counseling centers and IPV counseling centers increased. By 2001, more than 180 spousal violence counseling and support centers had been established in Japan. Counseling by these centers in 2007 numbered 62,078, about double the number in 2002 [18]. This indicates that given the opportunity, women in Japan seek help and receive support about IPV and that support has been expanding.

A systematic review concluded that there was evidence that IPV screening increased identification of women experiencing IPV, however, there was insufficient evidence of an effect for other outcomes such as recurrence of IPV or health measures [19]. Recently some evidence from antenatal care settings suggested that advocacy and empowerment interventions that followed IPV screening provided results in improved mental health outcomes of women [20, 21]. Based on this, WHO guidelines recommended IPV screening for women during their antenatal care [1]. In Japan, the Act on the Prevention of Spousal Violence and the Protection of Victims also stipulated the role of medical professionals for early detection and consultation. However, a survey of four prefectures in metropolitan areas found that IPV screening is still not widespread, with only 5% of institutions conducting IPV screening [22]. The common barriers to conducting IPV screening at health care settings have been reported as time limitations [23, 24], lack of confidence in screening [24, 25], difficulty to establish rapport [24], unease or fear about angering patients or causing emotional discomfort [26]. In a questionnaire survey of women in Japan who had experienced IPV screening, most women replied that it was not unpleasant [27, 28], but these were only a few questionnaire items answerable in yes/no format, to elicit the extent of satisfaction and with no psychometric controls applied. No other information was garnered about their experience.

Accordingly, this study aimed to conduct semi-structured interviews with postpartum women who received IPV screening during pregnancy to investigate women’s experiences of reading and completing the IPV screening questionnaire.

## Methods

### Design

The first part of this study was a descriptive IPV survey with a purposive sample of women. The second part of the study was qualitative using content analysis of semi-structured interviews of the same sample.

#### Study setting and participants

This study was conducted in a general hospital that provided antenatal to postpartum care in a city of the Nagano region. The city has approximately 28,000 people and is located near the Japanese Alps on the large island of Honshu. Participants eligible for this study were pregnant women whose due day was from the beginning of October through the end of November 2011 and planning on giving birth at the general hospital, and who were: (1) Japanese speaking, (2) had no severe complications and (3) able to participate in the informed consent process.

After approval by the Institutional Review Board of St. Luke’s College of Nursing (Approval No.: 11-039) the study commenced. Eligible women were invited to participate. Between September and December 2011, we consecutively recruited pregnant women face-to-face at their pregnancy checkup who matched inclusion criteria. We excluded one woman who was unable to read Japanese. IPV screening was conducted at the prenatal checkup at 35 weeks and onwards of pregnancy. Because at 35 weeks checkup women have an opportunity to consult with a midwife, we conducted IPV screening with self-administrated questionnaire at a privacy-secured room without her partner and other family members after midwife’s consultation. Participation was voluntary and participants were assured of their right to stop participating at any time without harm. They were also assured that their data would be kept confidential and anonymous.

#### Instruments

The screening instrument used was the VAWS [29]. VAWS is a 3-point Likert Scale comprised of seven items dealing with physical, psychological and sexual violence. Participants could respond: never = 0; sometimes = 1; or often = 2. A score of 2 points or more indicated IVP. Structural concept validity using factor analysis, and concurrent validity of the General Health Questionnaire and the Rosenberg Self Esteem Scale were established. Cronbach’s α was 0.70 for reliability. Kataoka [29] reported that when the Japanese version of the Index of Spouse Abuse (Japanese ISA) was applied as the optimized standard, sensitivity was 86.7% and specificity was 80.2%.

In addition to the VAWS, demographic information about the participants was also collected such as age, marital status, family configuration, educational background, employment status, annual income, parity, and information about the woman’s partner.

Researchers or a trained midwife distributed the self-administered VAWS questionnaire. Participants completed it at a location guaranteeing privacy. After the questionnaire was completed, researchers or a midwife from the hospital collected it.

#### Data collection

Maternity hospital stays are typically 5 – 7 days in Japan. Therefore allowing for physical recovery after childbirth, the interview to evaluate IPV screening during pregnancy was conducted on the 3^rd^ - 4th day after vaginal delivery, or the 5^th^ - 6th day after cesarean delivery during their hospital stay.

In order to obtain data of study participants’ experiences when reading and completing the VAWS, the researchers created an interview guide (see Additional file 1), to elicit participants’ opinions and thoughts concerning IPV screening, acceptability of the VAWS questionnaire items, such as words that participants found difficult to understand and expressions that participants found distasteful, and responses to screening methods and timing. Using the interview guide, the researchers conducted semi-structured interviews with participants for about 30 minutes. The interview contents were recorded on an IC recorder with the consent of the study participants. In order to confirm the interview contents and provide anonymity, ID numbers instead of individual names were used to identify questionnaires. One of the researchers (MI), a female nurse who was also a graduate student, visited the cooperating hospital and conducted the interviews. Before commencement of this study, researcher (MI) did pre-interviews whether she could interview according to the interview guide and listen to participant’s feeling and thought with non-judgmental manner. All interviews conducted after childbirth were at a location protecting the privacy of the survey participant and without the presence of the participant’s partner or other family members. Basically Interview was carried out once.

#### Follow-up of participants

Before starting this study, the researchers developed the support protocol for IPV victims at the hospital participating in this study and also informed the local Spousal Violence Counseling and Support Centers about the study. After IPV screening and the interviews, all participants in this study were provided information orally and in written form about social resources available in the region surrounding the cooperating hospital. Also, for women who screened positive and needed support, a trained midwife was appointed to liaise with this study and to provide consultation, safety planning and referral to the IPV support center in the community.

During this study, if a participant experienced some form of physical or mental problem or was judged to be at risk of IPV requiring specialist intervention, the researchers coordinated with university academics researching and advising about IPV, with advisors from Spousal Violence Counseling and Support Centers, and with the liaison midwife from the general hospital cooperating with this study, so that appropriate assistance could be provided with the safety of the women as first priority. Support was provided in accordance with the Perinatal Domestic Violence Support Guidelines [30]. If clear evidence of violence was discovered, after consent was obtained from the study participant, the midwife from the general hospital cooperating with this study reported the matter to the Spousal Violence Counseling and Support Center or the police.

#### Data analysis

Frequencies and percentage were used for quantitative data. Qualitative data were analyzed using content analysis [31]. The recorded contents from the IC recorder were transcribed literally. The transcriptions were read repeatedly, and divided up for words and phrases that captured the meaning of women’s experiences of IPV screening at the pregnancy checkup, and then labeled with codes that denoted the meaning. The codes were interpreted and compared based on differences and similarities. Codes were sorted into sub-categories, representing a more abstract level, and then subsumed into categories regarding their similarities. Finally, frequencies and percentages were calculated by categories. The second author (MI) coded each interview, and then discussed the coding with the first author (YK) until agreement was reached.

## Results

### Demographic characteristics of participants

Of the 48 women meeting the inclusion criteria during the study period and invited to participate in this study, agreement was received from 43 women (89.5%). The five not participating had been transferred to another hospital. After this information was provided and written informed consent was obtained data was collected. The valid response rate for the Violence Against Women Screen (VAWS) was 100%. All 43 women were then interviewed during their postpartum stay in the hospital.

Table 1 displays the demographic characteristics of participants. The majority (65.1%), of participants were in their thirties. Slightly over half were multiparas and the majority had vaginal births. All were married and most resided with their husbands. Family composition was nuclear family for 31 women (72.1%). Slightly over one-third of the participants were considered ‘housewives’ and the remainder worked full or part time. Most had graduated from high school and some had higher education. The couple’s annual income was between ‘$21,000-and $40,000 for half of the participants and between $41,000 and $61,000 or more’ for almost the other half. No one was receiving welfare payments. The participants’ spouses were generally in their thirties (67.4%) and most were employed (90.6%). One man (2.3%) was suspended from duty, and 3 men (7.0%) were unemployed.

**Table 1:** Demographic characteristics of participants and their partner (*N* = 43)

	n (%)
Participants	
Age (year)	
<20	1 (2.3)
20–29	14 (32.6)
30<	28 (65.1)
Marital status	
Married	43 (100)
Divorce history	
Wife	3 (7.0)
Husband	1 (2.3)
Living with partner	
Cohabitated	40 (93.0)
Separated	3 (7.0)
Family structure	
Nuclear families	31 (72.1)
Extended families	12 (27.9)
Educational background	
Junior high school graduate	4 (9.3)
High school graduate	10 (23.3)
Junior college graduate	17 (39.5)
University graduate / Graduate school	12 (27.9)
Employment status	
House duty	15 (34.9)
Full-time	16 (37.2)
Part-time	10 (23.3)
Others	2 (4.7)
Annual income (dollar)	
<200,000	2 (4.7)
200,000–400,000	22 (51.2)
400,000–600,000	9 (20.9)
600,000≦	9 (20.9)
Missing	1 (2.3)
Parity	
Primipara	19 (44.2)
Multipara	24 (55.8)
Partner of participants	
Age (year)	
20–29	9 (20.9)
30–39	29 (67.4)
40<	5 (11.6)
Employment status	
Full-time	38 (88.4)
Part-time	0 (0)
Suspension from work	1 (2.3)
Unemployment	3 (7.0)
Others	1 (2.3)

### Results of IPV screening in pregnancy

Table 2 indicates the frequency of responses for each question of VAWS during pregnancy. A total 37.2% of participants responded “sometimes” for the question “Is it difficult to settle by talking arguments between you and your partner?”, and 14% of participants responded “sometimes” for “feel frightened by their partner” and “Has your partner screamed and /or yelled at you?” “Sometimes” was answered by 4.7% for the question of “hit the wall or thrown object”. One woman responded “sometimes” for sexual violence, and also one woman responded “sometimes” for physical violence. There were 8 women (18.6%) who screened positive for IPV during pregnancy; in other words their VAWS score exceeded the cut-off of 2 points or greater and one was referred to the counseling center.

**Table 2:** Frequency of each question of VAWS during pregnancy

	Often	Sometimes	None
	n %	n %	n %
1. Is it difficult to settle by talking arguments between you and your partner?	0 (0)	16 (37.2)	27 (62.8)
2. Do you feel frightened by what he does or said?	0 (0)	6 (14.0)	37 (86.0)
3. Has your partner screamed and/or yelled at you?	0 (0)	6 (14.0)	37 (86.0)
4. Has your partner ever hit the wall or thrown objects?	0 (0)	2 (4.7)	41 (95.3)
5. Has your partner ever forced you to have sex?	0 (0)	1 (2.3)	42 (97.7)
6. Has your partner ever pulled your arm, pushed, slapped you?	0 (0)	1 (2.3)	42 (97.7)
7. Has your partner ever hit or kicked you?	0 (0)	0 (0)	43 (100)

### Women’s experiences of IPV screening

As a result of in-depth interviews, women’s experiences regarding IPV screening during pregnancy using the VAWS questionnaire were categorized into three themes: necessity, acceptability and optimality. The first theme ‘necessity’ included benefits for women through IPV screening. Three categories supported ‘necessity’: redefining the relationship, promoting IPV awareness, and opportunity to initiate support. The second theme was focused on women’s ‘acceptability’ of IPV screening, especially the VAWS questionnaire, and contained three categories: comfortable, quickly completed and difficulty. The third theme, ‘optimality’ referred to IPV screening during pregnancy that had appropriate timing for both women screening positive or negative. These three themes are discussed next in more detail.

#### Necessity

Participants talked about the necessity of IPV screening for all women during pregnancy. There were three categories under this theme: ‘redefining the relationship’, ‘promoting IPV awareness’ and ‘opportunity to initiate support’ which all indicated benefits of IPV screening for not only potential victims but also all pregnant women.

1. ‘Redefining the relationship’

There were 13 (30.2%) participants who discussed ‘redefining the relationship’. For example they expressed opinions such as, ‘It caused me to think about my relationship with my partner’. Participants were able to review their relationship with their partner and realized there were many positive attributes. There were also women who said that they felt the importance of support from their partner during pregnancy and child rearing. Of particular note was that this category included women who had screened positive on the VAWS during pregnancy. A few women (4.7%) said that IPV screening ‘provided an opportunity to discuss their relationship with their partner’. They had told their partner about the IPV screening, and used it as an opening to discuss their relationship.

‘Once again, I start to take a look at our relationship, and think how to create a good relationship between me and my husband.’

‘I thought about it (the relationship between us two), remembering the way of talking with my husband, times when we have had an argument by doing IPV screening,

(2) ‘Promoting IPV awareness’

There were seven women (16.3%) in the category ‘promoting IPV awareness’ due to IPV screening. These women said as a result of IPV screening, they understood that IPV was a serious social problem and that there was a large number of women troubled by violence and felt sympathy with them, They said ‘I became aware again about IPV’; ‘Women are burdened by troubles such as DV’.

‘From my opinion as a pregnant woman, women must never be subjected to it (violence). I have not experienced such violence, but if there are such women, they really are to be pitied. Question items (of VAWS) make me aware of it (violence) ’*.*

(3) ‘Opportunity to initiate support’

Three women (7%) said that IPV screening ‘provides an opportunity to receive support for women subjected to IPV’. In this category, the women indicated that IPV screening made disclosure easier and provided an opportunity to talk, and was linked to being able to get support. The participants said that particularly for women who had no one to consult with, this sort of opportunity was necessary.

‘If there are mothers worried by violence, it would be good if they can be provided support after giving birth. For women with no one to consult, it is an opportunity to talk about it’

#### Acceptability

Acceptability of using the VAWS questionnaire emerged from women’s experiences. There were three categories supporting acceptability: ‘comfortable’, ‘quickly completed’ and ‘difficulty’.

1. ‘Comfortable’

Most of the women, 42 women (97.7%), described some aspect of ‘comfortable’. In this category, a common expression was ‘it was not unpleasant’ or ‘I wasn’t particularly concerned’. This means women in general accepted being questioned about IPV and about the expressions used for the questionnaire items. Most women did not feel uncomfortable about IPV screening, and among the 8 women who were positive for IPV screening during pregnancy, 7 women replied ‘It was not unpleasant’.

‘Midwife promised that privacy was protected, so it wasn’t unpleasant’*.*

‘I haven’t experienced this problem, so I wasn’t concerned and uncomfortable. But I don’t know. Women who experienced violence would be concerned, I am not sure. I don’t feel uncomfortable for the questions (of IPV screening).’

(2) ‘Quickly completed’

There were 30 women (69.8%) who thought the VAWS screening tool was easy to read and answer. Participants commented: ‘There were no questions difficult to understand’, ‘I think it was easy to answer’. The VAWS is a 7-item screening tool and each item is short and simple; therefore women understood and found there were no items that were difficult to understand. Additionally, 40 women (93.0%) thought the IPV screening could be completed quickly, so it was ‘just right’ or ‘appropriate’. However, three women thought it was ‘a lot’.

‘There were no questions where I didn’t understand the intention of the question’

‘The same types of questions are repeated. If asking these questions at a pregnancy checkup, fewer questions are better’

(3) ‘Difficulty’

There were only 2 women (4.7%) who replied that ‘There was a question difficult to answer’. One of the reasons was that the contents of screening questions query about topics that are private especially regarding sexual violence. This woman screened negative in the VAWS test before pregnancy and during pregnancy. One woman responded that she did not feel discomfort about the questions, but thought that if her partner saw her answers he would feel uncomfortable, so they were difficult to answer. That woman screened positive in the VAWS test before pregnancy and during pregnancy.

‘There was a question difficult to answer a bit. That is about sexual violence. Because it is private matter. I can answer it, but I feel like difficult to answer’

‘I didn’t feel uncomfortable. But I feel the question is private. I answered yes to the question “Does your partner hit the wall or scream when he is angry”. If my husband looked at this answer, he would feel uncomfortable. I am afraid so.’

#### Optimality

The majority of the women (95.3%) thought that conducting IPV screening at the prenatal check-up was optimal. Those 41 women gave positive responses such as ‘I think it’s good’, and ‘I would not mind it’. On the other hand, 2 women gave negative responses such as, ‘It is not necessary’, and ‘I don’t think it is much good’. The reason for this was IPV is not an illness, so it is not necessary to deal with it at a hospital.

‘I think it’s good. It is helpful for women who have experienced violence’

‘I don’t think it is much good. Because IPV is different from an illness, so it is not necessary to deal with it at a hospital. There are other places to help women like shelters’*.*

In addition, when participants were asked whether they would consult with health care providers about IPV, only 8 women (18.6%) replied ‘Yes’. Reasons given for replying ‘Yes’ were ‘They seem close at hand’, ‘I want to consult a third party’, and ‘I can accept it if it is the opinion of a specialist’. Four women (9.3%) replied ‘I can’t say’. Reasons for replying ‘I can’t say’ were ‘I don’t know’, ‘It depends on the details’, and ‘It depends on the situation’. The majority, 31 women (72.1%), replied ‘No’. Reasons for replying ‘No’ were: ‘It is difficult to talk’, ‘I would talk with friends or family first’, ‘I would consult an IPV specialist’, ‘It would be a problem if my partner was informed of the fact that I consulted’, and ‘I don’t connect nurses with consultation about violence’.

## Discussion

Participants were about the same age, marital status and parity as most pregnant women in Japan [32]. The level of education and annual income of participants reflected the smaller cities and towns across Japan [33].

### Experiences of women who were screened for IPV during pregnancy

At the post-birth interviews for this study, concerning IPV screening using the self-completed VAWS questionnaire at a pregnancy checkup, 97% of participants replied that ‘They were not uncomfortable’. In earlier research, with similar populations, participants who replied that ‘They were not uncomfortable’ exceeded 80% in Kataoka’s study [27], and the rate was 97% in the study by Inami et al. [28], similar to the results of our study. These results indicate that IPV screening using VAWS questions can be answered without physical or mental burden on pregnant women, and without them feeling uncomfortable. Our study findings should eliminate concerns of health care providers such as fear of offending the woman or the woman’s reaction [26], and assist to promote the screening in prenatal settings in Japan. Moreover the VAWS is a self-administered questionnaire, so it can help women to feel secure. The RCT conducted in Japan [34] indicated that self-completed screening identified more abused women than face-to-face interviews. MacMillan et al. [35] also reported that women preferred self-completed approaches instead of face-to-face questioning. The VAWS questions and the self-completed questionnaire were the basic reasons why almost all participants felt comfortable in the study.

However a few women in our study were uneasy answering the questions. When the woman’s partner dominates her, the woman is deprived of feelings of personal control and feelings of safety, so may feel uneasy. The health care provider will develop a relationship where the woman feels safe. As indicated in the Feder, Hutson, Ramsay & Taket study, it is essential for all health care professionals to have a nonjudgmental, compassionate and sensitive attitude, and to maintain confidentiality [36]. Additionally, we found that there was a minority of negative opinions about the VAWS question items, even though the question contents can be considered to present low invasiveness for women. It is also necessary to consider revising VAWS, and refine simple question items so that they can be used amongst a busy medical environment.

### Benefits and possible problems for IPV screening

#### Benefits of IPV screening

IPV affects not only a woman’s health, safety and independence, but also affects the future of the child(ren). In addition, Petersen and colleagues [37] indicated that concern about children’s safety was a strong motivator for women to seek help or access services. Therefore, it is advantageous to conduct IPV screening at the location of perinatal care. Accordingly, in Japan where almost all women have health checkups by health care providers during pregnancy, conducting IPV screening at the location of perinatal care fulfills an important role in linking with interventions and continuing local support prior to the development into a serious problem. Conducting IPV screening provides an opportunity for women to disclose about intimate partner violence and an opportunity for health professionals to follow-up, and it can be linked to continuing support.

In addition, we found that IPV screening not only increased awareness concerning IPV, but also it triggered some women to think about their relationship with their partner. This may be considered a large benefit for the women. IPV screening provides a good opportunity for the women to review their relationship with their partner. Furthermore, among women victims, if the woman has not identified herself as being subjected to violence, the IPV screening may provide the opportunity for that recognition and awareness. In this study, a positive comment was received from a woman who was screening positive for IPV. Women who have been victims of IPV do not always perceive themselves as victims, thereby remaining isolated and alone. Awareness and naming of IPV for women can be considered the link towards recovery. Changing their self-perception may take time, however it is important from a long-term viewpoint, as they can share with other women who are IPV victims. By labeling the woman’s experience as IPV, the woman can finally join a support network [38].

#### Possible problems for IPV screening

There were several distinct problems found in this IPV screening. Systematic review of possible barriers of abused woman revealed one of the problems was fear of retaliation by the partner [26]. The partner may accompany the woman to the perinatal care location, and it may not be easy to guarantee privacy in a busy clinical situation [24]. In this study, some women worried if her partner knows the IPV screening result. One critical issue is to maintain privacy as much as possible and provide an environment where a woman can speak freely in peace. Next, the results of this study clarified that women do not associate IPV with hospitals, as a health care issue and they are not aware that nurses as health care providers can provide consultation about IPV. One factor for this lack of women’s awareness may be embedded in the health care environment itself due to health care providers’ low awareness about IPV. About 16 years have passed since enactment of the IPV Prevention Act. Guidelines for nurses were created [30] within several years after that. However, nurses who knew the contents of the DV Prevention Act represented less than 20% of nurses [39], and health care providers who have implemented countermeasures policy are still few [22]. One factor why DV screening has not spread was said to be that the screening method itself was not understood [22]. Other researchers pointed out that outcome evidence for conducting DV screening is insufficient and is a factor explaining why screening is not widespread [40, 41].

### Study limitations and future issues

It is possible that the small sample size and characteristics of participants and also location of this study was limiting. Accordingly, it is necessary to continue this study with diverse participants from a variety of backgrounds across a wider range of regions. Continued work on the questionnaire to eliminate unnecessary redundancy and confusing questions is also required.

## Conclusion

Evaluations by women who experienced IPV screening indicated that the majority did not find it uncomfortable. Although there were a few negative opinions about IPV screening, such as concern if their husband found out, interestingly among the positive opinions were comments about how the questions begin a fruitful reflective process about their marital relation plus it increased their awareness of IPV. IPV screening did not cause particular concern among women therefore health care providers should feel confident in including IPV education and screening for prenatal women. Future diffusion of IPV screening requires efforts to improve IPV awareness for health care providers, and provision of the required support environment that women can trust. This study clarified the evaluation of IPV screening from the women’s viewpoint. In order to support women’s health and safety, greater awareness about IPV by health care providers and promotion of IPV screening is required.

## Additional file

Additional file 1: Interview guide. (DOCX 17 kb)

## Abbreviations

IPV: Intimate partner violence
VAWS: Violence Against Women Screen
DV: Domestic Violence
