# Supplementary material for: Experiences of being screened for intimate partner violence during pregnancy: a qualitative study of women in Japan
Source: BMC Womens Health. 2018 May 29;18:75. doi: 10.1186/s12905-018-0566-4 (PMC5975515; doi:10.1186/s12905-018-0566-4)
Supplement: Supplementary file 1 — Interview guide. (DOCX 17 kb) [file 12905_2018_566_MOESM1_ESM.docx]

Interview Guide

1. What do you feel about IPV screening during pregnancy?

2. Did you feel uncomfortable?

If yes, Why? Where?

3. Were these any questions which you did not want to answer?

4. Were questions of the VAWS acceptable for you?

5. What did you think about environment that you get screened?

6. Did you feel secure when you answer the self-administrated questionnaire?

7. Were screening questions a lot?

8. Were there any questions that you couldn’t understand?

9. Do you want to consult with nurses about IPV?

10. Tell me about any opinions about IPV screening.
